# Supplementary material for: Comparative Proteomic Analysis Reveals the Regulatory Effects of H2S on Salt Tolerance of Mangrove Plant Kandelia obovata
Source: Int J Mol Sci. 2019 Dec 23;21(1):118. doi: 10.3390/ijms21010118 (PMC6981851; doi:10.3390/ijms21010118)
Supplement: Supplementary file 1 [file ijms-21-00118-s001.pdf]

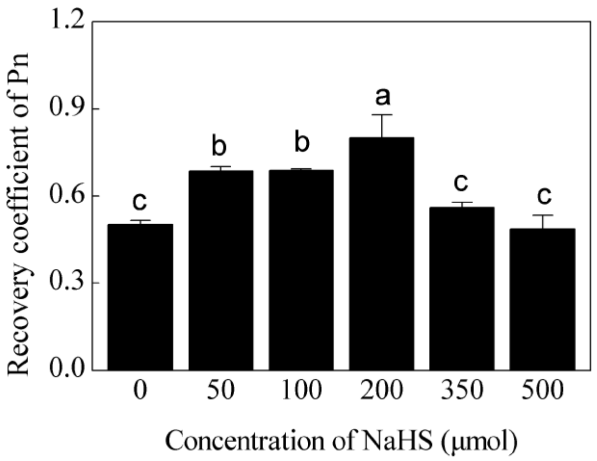

Supplementary Figure S1. Net photosynthetic rate (Pn) recovery coefficient of *K. obovata* seedling leaf under the treatments of series concentration of NaHS. Bars with different letters are significantly different at $P < 0.05$ according to Duncan's multiple range test.

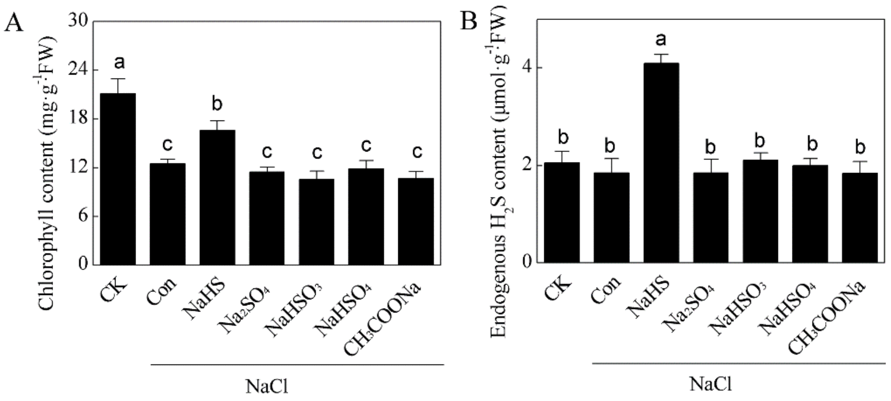

Supplementary Figure S2. H_2S and/or HS^-, but not other compounds derived from NaHS, contribute to (A) chlorophyll content and (B) endogenous H_2S accumulation. *K. obovata* seedlings were treated by 200 μM of NaHS, Na_2SO_4, $NaHSO_3$, $NaHSO_4$, or CH_3COONa, respectively, together with 400 mM NaCl for 7 days. The sample without any above sulfur-containing chemical but with 400 mM NaCl was regarded as the control (Con). CK represents the seedlings without NaCl and any above sulfur-containing chemical. Values are means ± SE of three independent experiments with at least three replicates for each. Bars with different letters are significantly different at $P < 0.05$ according to Duncan's multiple range test.

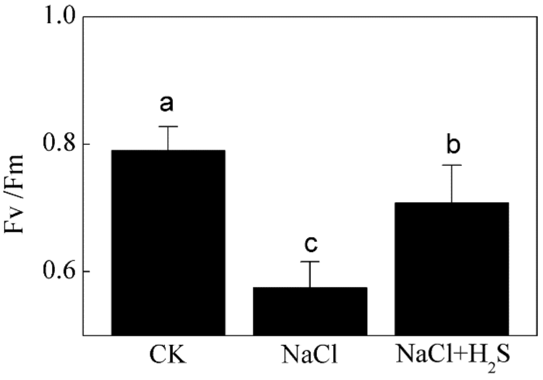

Supplementary Figure S3. Effects of NaHS on chlorophyll fluorescence characteristics of *K. obovata* seedling leaves. CK stands for the control which treated only by 1/4 strength Hoagland's nutrient solution. NaCl stands for 400 mM NaCl treatment. NaCl+H2S stands for 400 mM NaCl + 200 μM NaHS treatment. Error bars are SE (n=3). The columns labeled with different letters are significantly different at $P < 0.05$.

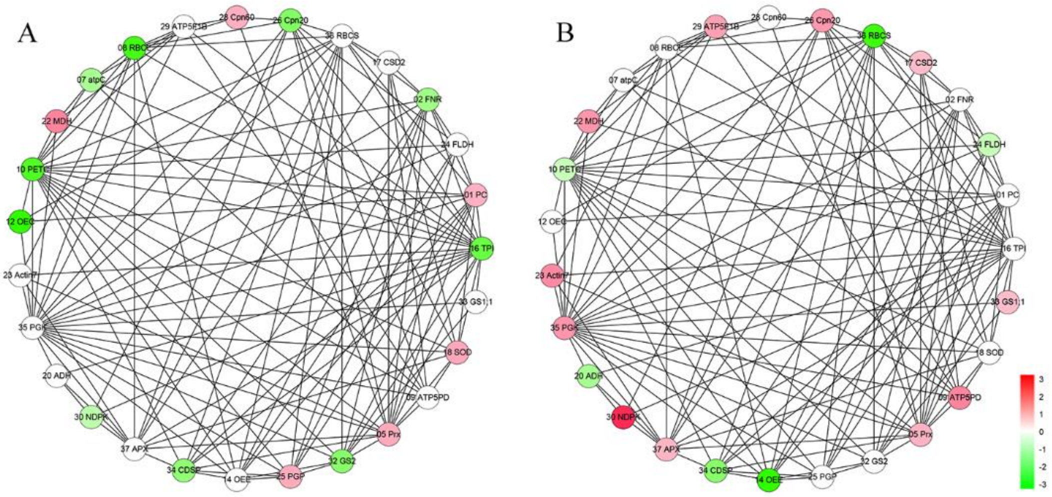

Supplementary Figure S4. Protein–protein interactions (PPIs) network analysis of 27 identified proteins from NaCl and NaHS treated *K. obovata* leaves. The association network was developed by STRING software using the homologous protein accessions from *Arabidopsis thaliana*. (A) the PPI network in NaCl vs CK, (B) the PPI network in NaCl+H2S vs CK. Nodes in red color represent up-regulated, green color represents down-regulated, white represent no change.

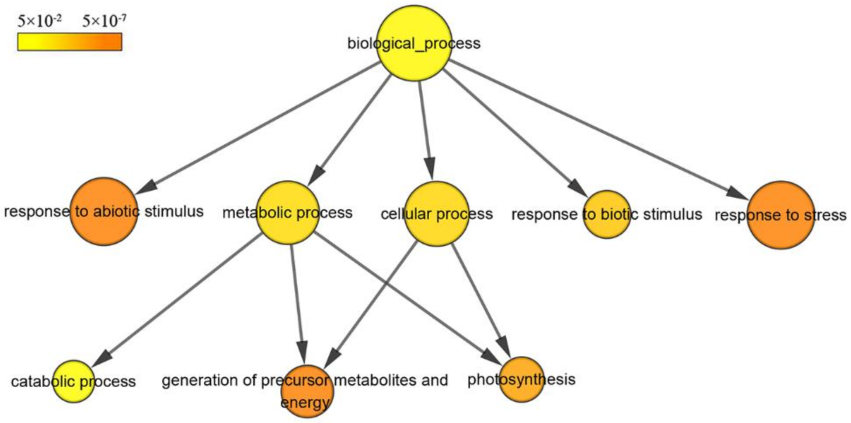

Supplementary Figure S5. Gene ontology analysis of the proteins identified from the leaves of *K. obovata*. Hierarchical directed acyclic graph in the aspect of biological process was performed. The yellow color represents the enriched GO terms.

Supplementary Table S1. Co-relations among different physiological indices in *K. obovata* leaves.

	**DW**	**Chl**	**Ci**	**Pn**	**Gs**	**Tr**
DW		0.379	0.042	0.500*	0.352	0.804**
Chl			-0.655*	0.895*	0.825**	0.406
Ci				-0.645**	-0.655**	-0.133
Pn					0.839**	0.695**
GS						0.469*

* Correlation is significant at the 0.05 level (2-tailed). ** Correlation is significant at the 0.01 level (2-tailed).

Supplementary Table S2. The detail information of the peptide sequence, expected and observed m/z values, sequence coverage for the different expressed proteins identified by MALDI-TOF/TOF MS in the leaves of *K. obovata* under the salinity and NaHS treatments.

Spot	Sequence[1]	Theoretical (Mr)[2]	Observed (Mr)[3]
	**Photosynthesis**		
1	NNAGFPHNVVFDEDEIPS GVDATK	17128.7	14050
2	LDFAVSR	40976.5	37320
	MYIQTR		
	DPNATVIMLATGTGIAPF R		
	ITGDDAPGETWHMVFST EGEIPYR		
8	EGNEIIR	50671.4	19870
	DDFIEKDR		
	EGNEIIREASK		
	TYYTPDYETK		
	LEDLRIPAAYAK		
	TFQGPPHGIQVER		
	GHYLNATAGTCEEMIK		
10	FYWAPTR	30594.1	58180
	EGPPEFEQPK		
	SFQCELVFAK		
	MCCLFINDLDAGAGR		
	LVNSREGPPEFEQPK		
	IVDSFPGQSIDFFGALR		
	MCCLFINDLDAGAGR		
12	VPFLFTIK	35412	36800
	RLTYEEIQSK		
	GGSTGYDNAVALPAGGR		
	DGIDYAAVTVQLPGGER		
14	STASVPIR	34802.9	34780
	VPFLFTIK		
	DGIDYAAVTVQLPGGER		
	FVEKDGIDYAAVTVQLPG GER		
36	FYWAPTR	52394	42290
	VPLILGIWGGK		
	EGPPEFEQPK		
	SFQCELVFAK		
	MCCLFINDLDAGAGR		
	IVDSFPGQSIDFFGALR		
	QVTSAVNYHGKSSNINR		
	**Carbohydrate and energy metabolism**		
6	RPGKPIETYLFAMFDENQK	37447.3	34330
7	QIIEANLALR	13694.4	14890
	LNGEWLTMALMGGFAR		
9	KFDDEIR	19781.1	18950

	TIDWDGMAK		
	FDDEIRNDYWGY		
	FSQEPEPINWEYYR		
16	FFVGGNWK	27497.2	23960
	KFFVGGNWK		
	CNGTTEEVKK		
	ALLNESNEFVGDK		
	SDFHVAAQNCWVR		
	ELAAQPDVDGFLVGGASLKPEFIDIIK		
	ELAAQPDVDGFLVGGAS		
	LKPEFIDIIKAATVK		
20	AWVYGEYGGVDVLK	41607.1	37230
	FDVVYDAIGQCDR		
	QFGSLAEYTAVEEK		
	EGGSVVALTGAVTPPGFR		
	GPFPFSQVAEAFSYIETNR		
	ENFEDLPEKFDVVYDAIGQCDR		
22	MAKEPMR	35846.3	40480
	ALGQISER		
	VLVVANPANTNALILK		
	FSSALSAASSACDHIR		
	KFSSALSAASSACDHIR		
	VLVTGAAGQIGYALVPMIAR		
	NVIIWGNHSSTQYPDVNHATVK		
24	MKPGFDPTK	36499	28240
	SDQFVTRGLVR		
	AEQYLADSGVPYTIIR		
	KAEQYLADSGVPYTIIR		
25	ISDFLSLK	40783.6	37320
	SQICMVGDR		
	IQYGTLCIR		
	VYVIGEDGILK		
	ISDFLSLKAAAV		
	NPLLSSNSAFLK		
	LVFVTNNSTKSR		
	ENPGCLFIATNR		
	GDSLIEGVPETLDMLR		
	EPLVVGKPSTFMMDYLANEFGISK		
29	MPNIYNALVVK	52293.2	57590
	TVAMSATDGLMR		
	AHGGVSVFGGVGER		
	FVQAGSEVSALLGR		
	VGLTALTMAEYFR		
	VALVYGQMNEPPGAR		
	DVNEQDVLLFIDNIFR		
	GMEVIDTGAPLSVPVGGATLGR		

	ELQDIIAILGLDELSEEDR		
	MPSAVGYQPTLSTEMGSLQER		
	IFNVLGEPVDNLGPVDTRTTFPIHR		
30	GLKLQNVEK	17034.8	16160
	SSQHNWIYEA		
	NVIHGSDSVESAR		
	EIALWFPEGIAEWR		
	ASEQTFIMIKPDGVQR		
35	NEPEFAK	50114	51050
	FSLAPLVPR		
	ASRAVVSMAK		
	VILSSHLGRPK		
	LVASLPEGGVLLLENVR		
	LASLADLYVNDAFGTAHR		
	**Amino acid and protein metabolism**		
4	NLAASVAGK	53517.9	40030
	DNPFGVKAMK		
	EVKNIYEEWR		
	AVANQPISVAIEAGGR		
	TPAKLHWPFGDQNK		
19	DAKAMASTPADVK	17808.1	16810
	VQVEDDNVLLISGER		
26	YAGNDFK	26898.4	23190
	IKIAEEK		
	DLKPLNDR		
	TAGGLLLTEATK		
	DLKPLNDRVLIK		
28	KLQTGVNK	65045.9	56090
	AEKEVLGQAAK		
	LADLVGVTLGPK		
	IAALKAPGFGER		
	VVAAGANPVQITK		
	GYISPYFVTDSEK		
	AAVEEGIVVGGGCTLLR		
	SQYLDDIAILTGGTVIR		
	LSGGVAVIQVGAQTETELK		
	SAENNLYVVEGMQFDR		
	KSQYLDDIAILTGGTVIR		
	LSGGVAVIQVGAQTETELKEK		
32	VGRDTEK	61638.1	44250
	AMVHRQMGQEVGNR		
	IIAEYIWIGGSGIDLR		
33	VGRDTEK	39318.5	40860
	EHIAAYGEGNER		
	HKEHIAAYGEGNER		
	IIAEYIWVGGSGMDMR		

	RPASNMDPYIVTSMIAETTILWNP		
	LTGHHETADINTFLWGVANRGASIR		
	**Hormone biosynthesis and transcription factor**		
13	SLKMEAK	35262.1	40100
	QDLNLVSK		
	AEEQCSQK		
	QHACPDMK		
	EELQRSNK		
	AEEQCSQKR		
	ILETSNESMR		
	KILETSNESMR		
	QHACPDMKEELQR		
	SFETLKQHACPDMK		
	RFLVFDQSGDQTTLLLASDIR		
21	WQIGISK	51008.1	44710
	MAVTSLTK		
	ILWKEPR		
	IEFLVNRMK		
	VLGMALGEMSR		
	FQIDPDGFLK		
	AVTSLTKATTLK		
	VEFFEGIGIPR		
	LVLYEIEEIEK		
	VFYLFPEVLGLDIGNR		
27	NMENAVADLTK	24115.5	23370
	AMAYHSGIGISK		
	LNSVLENQIFT		
	MAMAYHSGIGISK		
	LANEVKHLSSNR		
	LSDLIGDLQALVK		
	VLFIAVTGYTGTVLLK		
	VLFIAVTGYTGTVLLKNGK		
	VQDTVTTIHDDLSVVQHTVKMLDGR		
31	TTTGGGYGGGYGDSDTK	27332.2	39710
	HKIEEEVAAVAAVGSGGFAFHEHHEK		
	**Stress response proteins**		
5	SVDETKR	29144.1	21460
	SYGVLIPDQGIALR		
	EGVIQHSTINNLAIGR		
	SGGLGDLNYPLVSDVTKSISK		
	GLFIIDKEGVIQHSTINNLAIGR		
	TLQALQYVQENPDEVCPAGWKPGEK		
11	HVRSVMGDIR	33308.2	15980
	QAVTIPVMAKAR		
	VPADIRAQGGVAR		
	KIAAPYDLVMQTK		

	AQGGVARMSDPQLIK		
	AGTGVVAVYGNGAITETK		
	MAGTGVVAVYGNGAITETK		
	VGLAQMLRGGVIMDVVTPEQAR		
17	SAPLGQPFR	23291.9	19070
	GGHELSLTTGNAGGR		
	AFVVHELEDDLGK		
	AFVVHELEDDLGKGGHELSLTTGNAGGR		
18	EHGAPEDENR	15645.6	15020
	AVVVHADPDDLGK		
	FCVFVCVECGGAFPLF		
23	AGFAGDDAPR	41801.1	43650
	GYSFTTTAER		
	AVFPSIVGRPR		
	AEYDESGPSIVHR		
	IWHHTFYNELR		
	AEYDESGPSIVHRK		
	SYELPDGQVITIGAER		
	VAPEEHPVLLTEAPLNPK		
	DLYGNIVLSGGSTMFPGIADR		
34	LEASKTR	40911.9	27450
	GELIGEILR		
	VPHFSFYK		
	HCGPCVKVYPTVIK		
	DMDVIEVPTFLFIR		
	TCNDVEFILVMGDESEK		
37	EDKPEPPPEGR	27090.8	32520
	ELLSGEKEGLLQLPSDK		
	YAADEDAFFADYAEAHLK		
	**Unknown protein**		
3	SMEVVSGFR	54990.9	28300
15	GEEQEEAK	25734.2	29450
	FWVDYIDK		
	APLLPSDPYQR		
	DKAPLLPSDPYQR		
	CMQKESVAQSLADPK		

[1]The peptide sequence. C represent modification of carbamidomethyl, O represent modification of oxidation. [2]The theoretical m/z, mass over charge ratio of the parent ion.
[3]The experimental m/z, mass over charge ratio of the parent ion.

Supplementary Table S3. The modules of the protein-protein interaction networks and nodes biological process (GO) analysis.

Module name	Nodes	Edges	Cluster scores	Term ID	Term description	FDR	Matching nodes
A	9	32	8	GO: 0050896	response to stimulus	3.7×10^{-4}	CDSP, FLDH, FNR, GS2, OEE, PC, PETC, RBCS
B	8	22	6.286	GO: 0009628	response to abiotic stimulus	8.6×10^{-5}	APX, Cpn20, TPI, PGK, Prx, SOD

Supplementary Table S4. Sequences of forward and reverse primers used in qPCR for gene expression analysis in *K. obovata* leaves.

Spot number	Gene name	Annealing temperature	Forward primer sequence (5′ to 3′)	Efficiency(%)	R^2
18	*bHLH145*	59℃	F: GATGTTGCATGTGTTGGAGAAG<br>R: GAGAGAGATGTGTGGACTGAAAG	96.1	0.983
24	*SOD*	59℃	F: GCTTTGACCCAGGAAGATGA<br>R: CATTTGTTGTGTCGCCATACTC	98.7	0.980
25	*HSP*	59℃	F: GCAGGAGGAGAAGAACGATAAG<br>R: CCATGCTAGCTTTGACCTGATA	105.4	0.980
46	*OEE1*	59℃	F: AGGTGGCCTTGATTCTCAAATA<br>R: CAGCATGTATAGGCTCGGAAA	97.7	0.986
48	*Asr*	59℃	F: ATGACAGCAAGCCAAAGAGA<br>R: CTCCTACAGAGCGAGTGATAGA	93.9	0.9825
50	*GS1:1*	59℃	F: GAGACAACCATCCTCTGGAAAC<br>R: GTGGTGGAACTTAGGCAAACTA	103.3	0.999
	actin	59℃	F: AGCATCAGGCATCCATGAGAC<br>R: TGCTGAGAGATGCCAGAATG	94.7	0.986
